# Music in Churches

**Published:** 1855-03

**Authors:** Storrs Willis


### MUSIC IN CHURCHES.

BY STORRS WILLIS.

Worship, to my mind, implies an *act*. The nature of this act may best be expressed by the general word—homage. An act of homage may be rendered audibly and visibly, as accompanied by the voice and a corresponding posture of the body; or, it may be rendered silently and invisibly, unaccompanied by either voice or significant outward posture.

Homage is rendered the Supreme Being in *Praise*—in *Confession*—in *Petition;* also, as I conceive, in *Devout Meditation* on the divine works and attributes, or on one's own spiritual relations to his Maker—for, herein is a recognition of God, which is homage: and the homage we pay a Divine Being is of a quality necessarily involving worship. Worship, in its truest and highest sense, however, is when the soul ascends to the immediate presence of its God, and there pays him intelligent homage. It may be for a moment, like the upward glancing of a reverent thought from the crowded street of a city; or it may be for an hour, in solemn interview with the great Father.

It follows, then, that hearing a choir sing—is not worship; reading the hymn through in a merely intellectual attention to the thought—is not worship; a solemn feeling—is not worship. Such a feeling is often the result of architectural or artistic causes. A person, for instance, has entered a cathedral. He is awed by the grandeur and sacred hush of the place. He yields to an irresistible feeling of solemnity, and afterwards goes away and feels, perhaps, as though he had worshipped. Not so. He has merely indulged in what might be called *architectural awe.* Such a feeling is a legitimate effect of elevated art. But this is not yet worship. The place and the Supreme Object of worship lie higher than mere architecture or music, or sculpture, or painting, passively enjoyed, bear the soul. For, in the enjoyment of art, as in the enjoyment of natural scenery, we are recipients: the mind, therefore, is in a passive state. Whereas, in worship, the mind, as I contend, is in an active state. We must rise *through* nature to nature's God: and in sacred art, unless the soul be impelled forward one step further to definite religious action, it is not in a condition of worship:

for no passive state, no condition of mere feeling can involve this. Worship involves an act. Feeling may, and should, accompany this act, but cannot constitute it. Thus, in sacred song we must not only, in a mere act of intellection, acquire the thought of the words, but we must *utter that thought upward to God*—before we can be said rightly to worship.

In this manner only, as I can conceive, can the singing of a church choir ever become devotional to the exterior auditor. He may listen, enchanted, to the reiterated *Te Deums* of an extended service through all the churchly year, and yet not once have worshipped. Whereas, he may catch a single *Hallelujah*, or adoring aspiration, from the lips of the resounding choir, and, speeding it individually up from his own heart, though no sound have passed his lips, may have known an instant of true worship. Or, again, the pious eloquence of a devout organist may have so wrought upon the listener, through the mazes of solemn harmonies evolved on the majestic organ (beneath which were perceptible not only the skill of artistic fingers, but the throbbings of an earnest and religious heart), that he has been irresistibly impelled onward spiritually to exclaim, *Father, I adore thee!*—and music has preached effectively to his soul; for—he has worshipped.

In ordinary church service there are two acts of worship—the prayer and the music; the music, that is, in its ordinary accompaniment of the vehicles of intelligent thought—the psalms and the hymns.

This first act it is unnecessary to dwell upon; its nature is sufficiently distinct. The nature of the second act is much less clearly defined; for it is not, and cannot *always* be, worship; and this for the reason that *all* our psalms and hymns by no means embody the idea of worship. Some are a direct appeal to the Supreme Being, and are of this nature, being, in the strictest sense, prayers. But others are addressed to the audience; others to single classes of individuals; and others, still, are made the vehicles of precepts, doctrines, and other abstract teachings.

---

Longevity in Providence.—The following are the names of the persons who died in Providence during the year 1854, of the age of seventy years and upward. As usual, the number of females in the list largely preponderate. The fact is now more clearly established, as shown by every census, that after the age of seventy, the females exceed the males until the age of one hundred is passed, when the males again exceed the females:—

| | | | |
|---|---|---|---|
| John Howland - - | 97 | Elizabeth Shaw - - | 77 |
| Anne Kelley - - | 93 | Elizabeth A. Rhodes - | 77 |
| Mary Field - - - | 93 | Ann Drew - - - | 77 |
| Mary Dalson - - | 91 | Rachel Robinson - | 77 |
| Pardon Salisbury - - | 89 | Peter Langley - - | 76 |
| Bernard Whitney - | 89 | Mary Mason - - | 66 |
| Payton Dana - - | 88 | Bridget Gallagher - - | 76 |
| Nancy Todd - - | 88 | Charles McGirr - - | 76 |
| Nancy Jillson - - | 87 | Jane Johnson - - | 76 |
| Rosanna Dods - - | 87 | Sophia P. Balch - - | 75 |
| David Walker - - | 86 | Abraham Stillwell - - | 75 |
| John Smith - - | 86 | Moses Bartlett - - | 75 |
| Catharine Wise - - | 85 | Mary Justin - - - | 75 |
| Polly Stacy - - | 85 | Samuel Jackson - | 75 |
| John Murray - - | 84 | Catharine Hearn - - | 75 |
| Zelinda Weeden - | 84 | Joseph Dorr - - | 75 |
| Elisha Dyer - - | 83 | Eliza Lane - - - | 74 |
| Ann Sprague - - | 83 | Sarah A. Matthews - | 74 |
| Michael Walsh - - | 82 | Willam Brennan - - | 74 |
| Olive Brown - - | 82 | Nehemiah R. Knight - | 74 |
| Gideon Congdon - | 82 | Sarah Hill - - - | 74 |
| Elhanan W. Wade - | 82 | Elizabeth Bowen - | 73 |
| Adah Olney - - | 82 | Mary L. Potter - - | 73 |
| James McKenna - - | 82 | Sarah King - - | 73 |
| Elizabeth Wadsworth - - | 82 | Mary Stockman - - | 73 |
| Joseph Simmons - - | 82 | Mary Carpenter - | 72 |
| Dorcas T. Ashton - | 81 | Anna Waite - - | 72 |
| Joseph Smith - - | 80 | Elizabeth Corcoran - | 72 |
| Asenath Adye - - | 80 | Bridget Trainer - - | 72 |
| Sarah Haynes - - | 80 | Samuel N. Richmond | 72 |
| Margaret Ferguson - | 80 | David Barton - - | 71 |
| Mary Reed - - - | 80 | William Mayor | 71 |
| Phebe A. Babcock - | 80 | John S. Reynolds - | 71 |
| Sally Mason - - | 79 | Samuel Blake - - | 70 |
| Earl Potter - - | 79 | Mary Sollts - - - | 70 |
| Deborah Arnold - - | 78 | John Gerrin - - | 70 |
| Sarah Segur - - | 78 | John Easton - - | 70 |
| Nehemiah Scarborough - | 78 | Hannah Frank - - | 70 |

—*From the Providence Journal.*

